# Home Non-Invasive Ventilation Fails to Improve Quality of Life in the Elderly: Results from a Multicenter Cohort Study

**DOI:** 10.1371/journal.pone.0141156

**Published:** 2015-10-21

**Authors:** Adrien Tissot, Sandrine Jaffre, Frédéric Gagnadoux, Marc Levaillant, Frédéric Corne, Sylvaine Chollet, François-Xavier Blanc, François Goupil, Pascaline Priou, Wojciech Trzepizur, Antoine Magnan, IRSR NIV cohort group

**Affiliations:** 1 Service de pneumologie, L'institut du thorax, CHU Nantes, Nantes, France; 2 Département de Pneumologie, CHU Angers, Angers, France; 3 CNRS UMR8211-Inserm U988-EHESS, CERMES, Villejuif, France; 4 Service de Pneumologie, Centre Hospitalier du Mans, Le Mans, France; University of Bari, ITALY

## Abstract

**Background:**

Home non-invasive ventilation (NIV) is a widely used treatment for chronic hypoventilation but little is known on its impact in the elderly. In a multicenter prospective cohort study, we studied tolerance and efficacy of domiciliary NIV in patients aged 75 or more compared to younger ones.

**Methods and Results:**

264 patients with at least a six-month follow-up were analyzed. Among them, 82 were elderly. In the elderly and the younger, we found an improvement of arterial blood gas, the Epworth sleepiness scale and the Pittsburgh sleep quality index at 6 months. Mean daily use of NIV at 6 months was 7 hours and the rate of non-adherent patients was similar in both group. Health-related quality of life (HRQL) assessed by SF-36 questionnaires did not change significantly after NIV initiation in the elderly whereas HRQL improved in the less than 75. On univariate analysis, we found that diabetes was a predictive factor for non-adherence in the elderly (Odds ratio: 3.95% confidence interval: 1.06–8.52).

**Conclusion:**

NIV was efficient in the elderly while evaluation at 6 months showed a good adherence but failed to improve HRQL.

## Introduction

Long-term non-invasive ventilation (NIV) is a widely used treatment for chronic respiratory failure (CRF) in developed countries. Validated indications are restrictive disorders and obesity-hypoventilation syndrome (OHS) for which previous studies provided proofs of efficacy on arterial blood gas (ABG) parameters, hospital stays and health status [1–3]. NIV is also widely used in Europe in patients with chronic obstructive pulmonary disease (COPD), albeit this indication remains controversial [4]. This treatment requires the cooperation and acceptance of the patient to insure compliance and efficiency. Due to high rate of disability and functional decline in elderly patients [5], cooperation can be difficult and therefore NIV denied to this population. However, last decades have seen a remarkable increase in the number of patients over 75 years old treated with NIV in Europe. For example, these patients now represent more than 40% of ventilated adults in France, compared to 30% in 1996 [6]. Data from the US health and retirement study showed 12.3% of chronic lung disease patients as being aged ≥ 65 years old [7]. Despite the fact that elderly patients now represent a large part of the severe CRF population, very few data on NIV adherence and efficiency are available in this specific population. Clinical practice guidelines [8,9] recommend looking for motivation of both the patient and the family and do insist on a comprehensive education but do not mention any specific issue in aged patients.

In this multicenter prospective cohort study, we aimed to evaluate the efficacy of long term NIV on ABG values, sleep quality and health-related quality of life (HRQL) in elderly patients as compared to the younger ones.

## Materials and Methods

### Study population

This prospective cohort was conducted in three hospitals from the West of France (Angers, Le Mans and Nantes). Since September 2009, consecutive patients in whom NIV was prescribed for CRF have been recruited in the *Institut de Recherche en Santé Respiratoire (IRSR) NIV cohort*. All patients ≥ 18 years old and able to give informed consent were eligible. For this study, we extracted the cohort data in February 2014 and analyzed every patient with at least 6 months of follow-up or who died before 6 months. Approval was obtained from the University of Angers ethics committee (2009/17) and from the Comité Consultative sur le Traitement de l’Information en matière de Recherche dans le domaine de la Santé (07 207 bis). The database is anonymous and complies with the restrictive requirements of the Commission Nationale Informatique et Liberté (908157), the French information technology and personal-data protection authority. All patients gave their written informed consent at enrolment.

### Patients' schedule

After inclusion in the cohort, patients underwent a baseline evaluation including ABG, recording of patient characteristics (age, gender, body mass index (BMI), marital status, smoking habits), etiology of CRF and associated diseases. Patients were classified as having cardiovascular comorbidities if they reported at least one of the following: known and treated hypertension, ischemic heart disease, cardiac arrhythmia, congestive heart failure and stroke. They were followed-up with a medical visit 4 months after initiation of NIV, one year after and then every year. Home routine visits were carried out by a health care provider every 3 months and the ventilation adherence was assessed by records of mean daily use of ventilation.

### Standardized scores and scales used in the study

Subjective daytime sleepiness and quality of sleep were evaluated by the Epworth sleepiness scale (ESS) [10] and the Pittsburgh sleep quality index (PSQI) [11], respectively. Cognitive state was evaluated by the Mini mental state examination (MMSE) [12]. Health status was assessed by the Medical Outcome Survey 36-items Short-Form Health Survey (SF-36) and expressed with the physical component summary (PCS), the mental component summary scales (MCS) and subscales [13].

### Definition of patients' profile

Three groups were considered according to the profile of CRF: restrictive disorders, OHS and obstructive disorders. Restrictive disorders included neuromuscular diseases, chest wall abnormalities, post-tuberculosis or chest surgery sequelae. OHS was defined by both BMI ≥ 30 kg/m^2^ and daytime hypercapnia (PaCO_2_ > 45 mmHg) in the absence of any other cause of hypoventilation [14]. Obstructive disorders included COPD and bronchiectasis. Patients were defined as NIV-adherent if they were still using NIV at 6 months with a mean daily use of at least 4 hours per night. Non-adherence corresponded to patients who refused NIV, who had stopped treatment or who were using it for an average of less than 4 hours per night. We used last available data of adherence for patients who died before 6 months of follow up.

### Statistical analysis

Statistical analysis was performed with SAS software (SAS/STAT Package 2002–2003 by SAS Institute Inc., Cary, NC, USA). Characteristics at baseline of patients aged 75 and more (≥ 75) were compared with the ones of patients whose age was less than 75 (< 75) using Chi-square test for categorical variables and 2-sample t-test for continuous variables. A logistic regression procedure was conducted to determine the factors of treatment adherence. Results were expressed as percentages, mean (standard deviation) (SD) and odds ratio (OR) (95% confidence interval). A 2-tailed p value < 0.05 was considered significant. Paired t-test was used to compare ABG, observance and SF-36 at baseline and after 6 months of follow-up.

## Results

### Study population

Between September 2009 and February 2014, 264 patients were enrolled in our cohort. Mean time of follow-up was 21.8 months (range 0 to 52). All these patients were on a bi-level airway pressure regimen. Among them, 82 (31%) were aged ≥ 75 while the remaining 182 (69%) were < 75. Table 1 gathers baseline data for these two groups. Comparison between the < 75 and the ≥ 75 demonstrated significant differences for cardiovascular comorbidities, etiology distribution and smoking habits. There was a higher percentage of patients with OHS in the elderly group (52% vs. 37%, p = 0.02). Patients < 75 had less associated cardiovascular comorbidities (87% vs. 61%, p < 0.0001) and had more frequently a smoking history. No significant difference was observed between the two groups in terms of gender, BMI, long-term oxygen therapy, pulmonary function test and NIV settings.

**Table 1:** Study population at baseline.

	≥ 75 (n = 82)	< 75 (n = 182)	p value
Women	38 (46)	76 (42)	0.7
BMI, kg/m^2^	35 (10)	36 (13)	0.4
Cardiovascular comorbidities	71 (87)	111 (61)	<0.0001
Smokers	32 (39)	107 (59)	0.003
LTOT	23 (28)	44 (24)	0.4
Obstructive disorder	19 (23)	56 (31)	0.2
Restrictive disorder	18 (22)	53 (29)	0.2
OHS	43(52)	67 (37)	0.02
NIV initiation in ICU	27 (33)	69 (38)	0.4
FEV_1_, % of predicted	66 (22)	64 (24)	0.6
FVC, % of predicted	71 (20)	73 (20)	0.6
FEV_1_/FVC, %	70 (18)	70 (16)	0.8
IPAP, cm H_2_O	19.9 (2)	19.9 (4)	0.9
EPAP, cm H_2_O	8.1 (3)	7.7 (2)	0.4
Min RR	14.6 (2)	14.9 (2)	0.3
Full-face mask	69 (84)	157 (86)	0.7

Values expressed as mean (SD) or n (%). BMI = body mass index, LTOT = long-term oxygen therapy, OHS = obesity hypoventilation syndrome, ICU = intensive care unit, FEV_1_ = forced expiratory volume in 1 s, FVC = forced vital capacity, IPAP = inspiratory partial airway pressure, EPAP = expiratory partial airway pressure, Min RR = minimal respiratory rate

### Predictive factor for adherence in the elderly

Mean daily use of NIV at 6 months was 7 ± 3 hours for the ≥ 75 and 7 ± 4 hours for the < 75 (p = 0.6). We found 11 patients ≥ 75 (13%) and 14 patients < 75 (8%) who quitted NIV for any other cause than death (p = 0.2). Non-adherent patients were 28 (34%) in the elderly and 47 (26%) in the younger. There was no significant difference between the two groups regarding adherence (p = 0.7). Univariate analysis taking into account age, gender, BMI, etiology, diabetes, cardiovascular comorbidities, smoking habit, marital status, mask type and MMSE score (threshold = 25) only showed a significant impact of diabetes on compliance in the elderly (p = 0.02) (Table 2). Results of multivariate analysis confirmed that only diabetes was predictive of non-adherence.

**Table 2:** Predictive factors for adherence in patients aged ≥ 75.

	Odds ratio	95% Confidence interval	p value
Age	0.93	(0.83–1.03)	0.1733
Women	1.39	(0.53–3.64)	0.5092
OHS	0.97	(0.92–1.03)	0.3277
Obstructive syndrome	1.25	(0.41–3.81)	0.6963
Restrictive syndrome	1.91	(0.63–5.76)	0.2507
Diabetes	3.01	(1.06–8.52)	0.0377
Cardiovascular comorbidities	1.56	(0.41–5.95)	0.5117
Smoking habit	1.29	(0.47–3.51)	0.6234
Living in couple	1.44	(0.55–3.83)	0.4596
MMSE ≥ 25	2.61	(0.91–7.51)	0.2806

OHS: obesity hypoventilation syndrome, MMSE: mini mental state examination

### Comparison between initial and 6 month visits

Among the 264 patients described above, 11 died and 25 stopped treatment before 6 months while data was missing data for 69 patients. Overall, we found 159 patients with a complete 6 months follow-up for the assessed values. For patients ≥ 75, initial ABG showed a high PaCO_2_ value over 60 mmHg and very low hypoxemia consistent with severe CRF. Initiation of NIV enabled a global improvement of the PaCO_2_ level, which decreased below 45 mmHg at 6 months (p < 0.0001). PaO_2_ improved as well, rising up significantly. We found a significant improvement for both absolute values of ESS and PSQI and a marked reduction of the number of patients with initial abnormal score (Table 3). The initial MSC was 46.9 ± 6 and PCS was 49.4 ± 3. Neither of these two scores improved significantly at 6 months (Table 4 and S1 Table). Looking at the subscales, we found an improvement in the sole emotional role scale.

**Table 3:** Comparison of NIV efficacy at 6 months in patients aged < 75 years or ≥ 75 years.

	≥ 75 years (n = 44)			< 75 years (n = 115)			p value^‡^
	Baseline	At 6 months	Δ	Baseline	At 6 months	Δ	
ESS: mean (SD)	8.8 (5)	4.9 (3)	-4.3 (5)*	8.2 (5)	5.2 (4)	-3.2 (4,)*	0.32
ESS over 10: %^†^ (SD)	27.8 (45)	3.4 (19)	-26.1 (45)*	37.4 (49)	10.5 (31)	-28.4 (48)*	0.83
PSQI: mean (SD)	8.4 (4)	5.2 (3)	-2.1 (3)*	9 (4)	5.7 (3)	-3.5 (5)*	0.11
PSQI over 7: % ^§^ (SD)	87.9 (33)	52.2 (51)	-29.4 (59)	82.9 (38)	55.4 (50)	30 (54)^†^	0.95
PaO_2_: mean (SD)	63.2 (15)	69.5 (9)	6.5 (16)*	66.9 (13)	75.8 (14)	8 (17)*	0.66
PaCO_2_: mean (SD)	61.7 (13)	42.9 (5)	-20.6 (15)*	56 (10)	42.1 (5)	-14.8 (12)*	0.04

Δ: variation between baseline and 6 months

*p value < 0.05

^†^percentage of patients with initial Epworth scale score over 10

^§^ percentage of patients with initial Pittsburgh sleep quality index over 7

^‡^p value for comparison of Δ between ≥ 75 and < 75

ESS: Epworth scale score, PSQI: Pittsburgh sleep quality index

**Table 4:** Comparison of the impact of NIV on HRQL evaluated by SF-36 in patients aged < 75 years or ≥ 75 years.

	≥ 75 years (n = 44)			< 75 years (n = 115)			
	Baseline	At 6 months	Δ	Baseline	At 6 months	Δ	p value^‡^
Physical functioning	28.1 (22)	32.2 (27.5)	1.5 (26)	37.6 (24)	48.8 (31)	11.2 (29)*	<0.0001
Role: physical	23.4 (34)	45.7 (42)	18.1 (49)	19.8 (33)	60.4 (43)	26.3 (40)*	<0.0001
Body pain	57.0 (32)	57.8 (28)	-5.5 (46)	47.7 (28)	57.9 (30)	7.9 (40)*	0.03
General health	44.7 (20)	47.7 (18)	2.4 (21)	43 (20)	49.1 (22)	4.5 (20)*	0.02
Vitality	42.9 (24)	50.6 (20)	5.5 (32)	38.1 (23)	55.6 (24)	17.2 (30)*	<0.0001
Social functioning	68.1 (31)	76.4 (30)	1.8 (33)	54.5 (29)	74.7 (27)	16.6(36)*	<0.0001
Role: emotional	46.2 (44)	66.2 (39)	24 (56)*	37.1 (41)	73.8 (39)	33.2 (53)*	<0.0001
Mental health	62.1 (22)	67.5 (18)	0 (24)	53.9 (23)	65.9 (22)	9 (23)*	0.0003
PCS	49.4 (3)	49.2 (2)	0.1 (3)	50.3 (3)	49.8 (2.4)	-0.7 (3)*	0.04
MCS	46.9 (6)	48.4 (5)	0.3 (7)	44.7 (7)	47.7 (6.5)	3.6 (7)*	<0.0001

Values expressed as mean (SD).

Δ: variation between baseline and 6 months

*p value < 0.05

^‡^p value for comparison of Δ between ≥ 75 and < 75

PCS: physical component summary, MCS: mental component summary

For the group < 75, PaCO_2_, PaO_2_, ESS and PSQI were significantly improved at 6 months. We found an increase of the MCS with an initial value of 44 ± 7 and a 6 months value of 47.5 ± 7 (p < 0.0001). All the 8 subscales of the SF-36 improved as well. We also found a decrease of the PCS with an initial value of 50.6 ± 3 and a 6 months value of 49.9 ± 2 (p = 0.0008) (S2 Table).

When we analyzed the difference of variations between the initial visit and the 6 month visit, the PaCO_2_ decrease was more pronounced in the ≥ 75 group as compared to the < 75 group (p = 0.04). The SF-36 improved only in the < 75 whereas it was stable in the ≥ 75. Regarding the PaO_2_, the ESS, the PSQI and the PCS, evolution at 6 months was similar between the two groups (Table 4).

## Discussion

In this cohort study, NIV was found to be efficient in the elderly, as indicated by markedly improved ABG values and sleep quality scores. However, the elderly seemed to have less benefit in terms of quality of life. Diabetes was found to be a predictive factor for non-adherence.

To our knowledge, this is the first prospective study evaluating domiciliary NIV in the elderly population. Data indicate a good adherence and efficacy in this specific population. Daytime ABG improved remarkably with home NIV, as attested by the mean PaCO_2_ value at 6 months, which was 42.9 mmHg. This is particularly important to notice as both Tsuboi et al. and Marti et al. recently showed that a good control of PaCO_2_ level was a predictor of survival in a population of patients with restrictive disorders [15,16]. We managed to reach a lower mean PaCO_2_ than in these two studies, probably because we used a relatively high ventilation pressure. Interestingly, high intensity ventilation was not associated with a HRQL alteration in our study. Moreover, it has been shown that high IPAP (28.6) as compared to low IPAP (14.6) was associated with a better compliance and a better efficiency in COPD patients [17]. The greater improvement seen in our elderly population was probably related to a greater number of patients with OHS as compared to the younger part of the cohort. In this etiology, NIV has been proved to be highly effective in normalizing hypercapnia [3,18]. In a monocentric retrospective study of 43 patients aged 75 years or more, Farrero et al. also found that NIV was efficient with a significant improvement in ABG 6 months after NIV initiation [19]. A smaller study on 6 elderly patients found NIV to be efficient on ABG [20]. Our study also demonstrated the positive impact of home NIV in sleep quality, as assessed by the Epworth scale score and the Pittsburgh sleep quality index. However, one limitation is that we did not perform any polysomnographic recording, as our main objective was to evaluate HRQL and tolerance. Our elderly population presented a high compliance on ventilator with a mean daily use of more than 6 hours, which is comparable to data from larger studies on domiciliary NIV [3,21]. In the two studies evaluating domiciliary ventilation in the elderly, good compliance was found with a mean daily use of 8.3 and 10.5 hours [19,20]. Only 5 patients (11%) discontinued treatment in the Farrero study. In our study, we found a higher percentage of discontinuation in the elderly (14%). However, we did not find significant differences compared with the younger part of our cohort. Thus, in a selected population of old patients, it seems that compliance is not an issue.

Surprisingly, diabetes seemed to be a predictive factor for non-adherence in the elderly. We can only speculate that the addition of NIV was hardly accepted by these patients already treated with oral or injectable treatment, but we do not have a clear explanation for this finding. Further studies specifically addressing this issue are clearly needed.

An initial MMSE score below 25 did not impact adherence in the older part of our population. Lower MMSE threshold did not appear to interfere with compliance as well (data not shown). This finding has to be tempered by the low number of patients with severe cognitive impairment in our cohort (MMSE < 20, n = 7). It is likely that patients with severe cognitive impairment and with no helping relatives were not easily proposed long term NIV and were therefore not included in our study. We might have underestimated the role of age, cognitive function and social interactions for long term NIV adherence.

With the exception of its emotional subscale, we did not find any improvement of the SF-36 questionnaire following NIV initiation in the elderly whereas domiciliary NIV improved all subscales and the mental component summary in patients < 75. This could be a limitation to consider when initiation of long term NIV is discussed in an elderly patient. The wellbeing and the quality of life of these specific patients are key targets for this treatment. However, these outcomes have never been reported in an old patient sample. Thus, more studies are clearly needed to confirm or disprove our findings. Furthermore, we must questioned the evolution of HRQL in old patients who are not treated with domiciliary NIV. An hypothetical controlled study conducted in an elderly population with chronic hypercapnia treated or not by NIV could help to answer such a question but is of course very unlikely to happen because of obvious ethical considerations.

Windisch et al. studied the impact of home mechanical ventilation on HRQL in 85 patients with younger age and various etiologies for CRF [22]. They found a significant increase of the SF-36 PCS and MCS at one and twelve months following NIV establishment. The best results were observed in patients with OHS or restrictive thoracic disorder. For this remarkable difference between the elderly and the younger patients, we speculate that associated comorbidities, which are more frequent in the elderly, altered the HRQL improvement. We found that cardiovascular comorbidities were more frequent in our elderly population. It is well known that chronic diseases and physical disabilities are more important with age [7,23].

We conclude that domiciliary NIV was an efficient and well-tolerated treatment for severe CRF in the elderly. However it failed to improve HRQL. These results do not challenge initiation of home NIV in the elderly but engage a closer follow-up with reassessment of its benefit at one point to pursue or discontinue the treatment.

## Supporting Information

S1 Table Evolution of HRQL after NIV initiation in patients aged ≥ 75 (n = 44) (DOCX)

S2 Table Evolution of HRQL after NIV initiation in patients aged < 75 (n = 115) (DOCX)
