# Supplementary material for: Home Non-Invasive Ventilation Fails to Improve Quality of Life in the Elderly: Results from a Multicenter Cohort Study
Source: PLoS One. 2015 Oct 21;10(10):e0141156. doi: 10.1371/journal.pone.0141156 (PMC4619542; doi:10.1371/journal.pone.0141156)
Supplement: S1 Table — (DOCX) [file pone.0141156.s001.docx]

S1 Table. Evolution of HRQL after NIV initiation in patients aged > 75 (n = 44)

|  | Baseline | At 6 months | p |
| --- | --- | --- | --- |
| Physical functioning | 28.1 (22) | 32.2 (27.5) | 0,8 |
| Role: physical | 23.4 (34) | 45.7 (42) | 0.06 |
| Body pain | 57.0 (32) | 57.8 (28) | 0.5 |
| General health | 44.7 (20) | 47.7 (18) | 0.6 |
| Vitality | 42.9 (24) | 50.6 (20) | 0.4 |
| Social functioning | 68.1 (31) | 76.4 (30) | 0.8 |
| Role: emotional | 46.2 (44) | 66.2 (39) | 0.03 |
| Mental health | 62.1 (22) | 67.5 (18) | 1.0 |
| PCS | 49.4 (3) | 49.2 (2) | 0.8 |
| MCS | 46.9 (6) | 48.4 (5) | 0.8 |

Values expressed as mean (SD). p value for comparison between baseline and 6 months. PCS: physical component summary, MCS: mental component summary
