# Supplementary material for: Home Non-Invasive Ventilation Fails to Improve Quality of Life in the Elderly: Results from a Multicenter Cohort Study
Source: PLoS One. 2015 Oct 21;10(10):e0141156. doi: 10.1371/journal.pone.0141156 (PMC4619542; doi:10.1371/journal.pone.0141156)
Supplement: S2 Table — (DOCX) [file pone.0141156.s002.docx]

S2 Table. Evolution of HRQL after NIV initiation in patients aged < 75 (n = 115)

|  | Baseline | At 6 months | p |
| --- | --- | --- | --- |
| Physical functioning | 37,6 (24,2) | 48,8 (31,0) | <0,0001 |
| Role: physical | 19,8 (32,8) | 60,4 (43,0) | <0,0001 |
| Body pain | 47,7 (27,6) | 57,9 (30,3) | 0,03 |
| General health | 43,0 (20,1) | 49,1 (22,5) | 0,02 |
| Vitality | 38,1 (22,8) | 55,6 (24,0) | <0,0001 |
| Social functioning | 54,5 (28,6) | 74,7 (26,7) | <0,0001 |
| Role: emotional | 37,1 (41,2) | 73,8 (39,1) | <0,0001 |
| Mental health | 53,9 (22,9) | 65,9 (22,4) | 0,0003 |
| PCS | 50,3 (2,8) | 49,8 (2,4) | 0,04 |
| MCS | 44,7 (6,7) | 47,7 (6,5) | <0,0001 |

Values expressed as mean (SD). p value for comparison between baseline and 6 months. PCS: physical component summary, MCS: mental component summary
